# Possible Toxin-Induced Acute Necrotising Encephalitis (ANE) With Secondary Vasculopathy and Paroxysmal Autonomic Instability With Dystonia (PAID) Syndrome

**DOI:** 10.7759/cureus.50100

**Published:** 2023-12-07

**Authors:** Kasim Aslam, Hasaam Uldin, Laura Smith

**Affiliations:** 1 Rehabilitation Medicine, Moseley Hall Hospital, Birmingham, GBR; 2 Radiology, University Hospitals Coventry and Warwickshire, Coventry, GBR

**Keywords:** acute encephalitis, methylprednisolone, acute necrotizing encephalitis, cytokine storm syndrome, paroxysmal autonomic instability with dystonia

## Abstract

Acute necrotising encephalitis (ANE) is a rare and life-threatening disorder typically associated with viral pathogens triggering an inflammatory response. It is characterised by rapid neurological deterioration linked to a cytokinetic storm which radiologically manifests with cerebral radiological changes. We present a unique case not previously documented of an immunocompetent 23-year-old male who survived the course of ANE, with widespread involvement of the brain including the deep white matter, cortex, superior frontal gyrus, occipital lobe and cerebellum. His disease course was complicated by a ventilator-associated empyema, paroxysmal autonomic instability with dystonia (PAID) syndrome) and livedo reticularis which cumulatively resulted in a poor neurological outcome.

## Introduction

Acute necrotising encephalitis (ANE) is a rare disease typically associated with viral pathogens, such as influenza, herpes simplex virus, mycoplasma and human herpesvirus-6 [1]. Although these viruses are the usual precipitants, the disease manifests due to the resultant high levels of proinflammatory cytokines including tumour necrosis factor receptor-1, interleukin-1, and interleukin-6, the levels of which determine the disease’s severity [2]. The affected demographic is usually infants and children from the far-east region such as Taiwan and Japan [3]. However, with the increased use of imaging, cases have appeared worldwide with a more recent increase due to COVID-19 having an association with neuroinflammation [4]. We present a unique case not previously documented of an immunocompetent 23-year-old male who survived the course of ANE, with no detected viral cause but positive tetrahydrocannabinol (THC) metabolites but was consequently diagnosed with paroxysmal autonomic instability with dystonia (PAID) syndrome and livedo reticularis.

## Case presentation

A previously healthy 23-year-old male presented to the emergency department with a one-week history of a productive cough, agitation, disorientation and slurred speech with a worsening headache over the course of the previous day. On initial examination, he had a Glasgow Coma Scale (GCS) score of 12/15, yet an abbreviated mental test score (AMTS) of 2/10 with associated visual changes. Specifically, this caused asymmetrically bilateral reduced vision predominately in the left eye, as well as vertical and right-sided nystagmus. He demonstrated hyperreflexia in the biceps, triceps, knee and Achilles with hypertonia in all 4 limbs. He was unable to walk with poor proprioception and had an exaggerated jaw reflex with Babinski’s test abnormal bilaterally.

**Figure 1:**
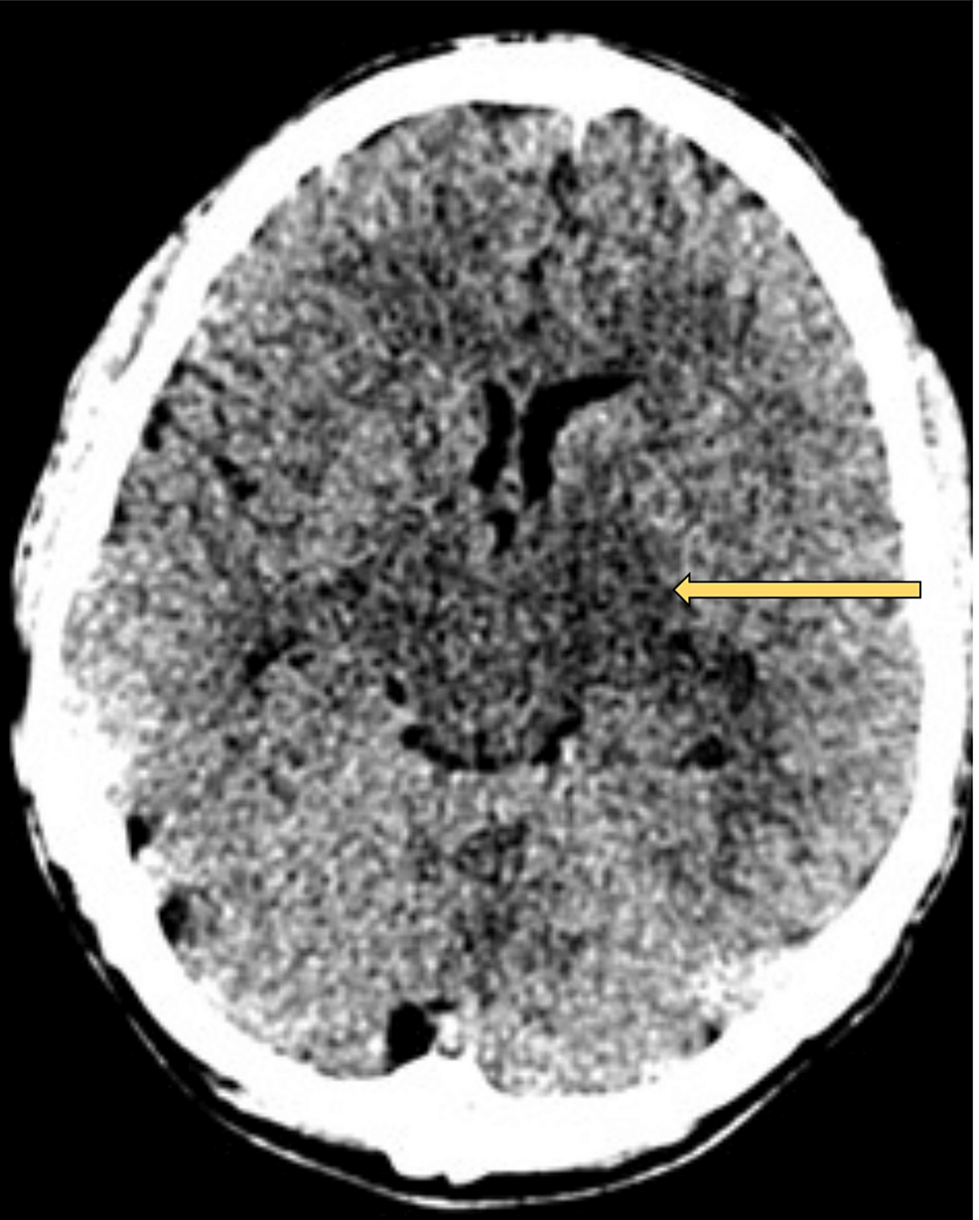
Hypodensity noted in bilateral thalami, more so on the left side (yellow arrow); the midbrain also demonstrating similar homogenous hypodensity

Our initial concern was meningoencephalitis and we started empirical treatment with IV antibiotics, antivirals and dexamethasone. The initial CT (Figure 1) demonstrated an asymmetry appearance of the grey-white matter differentiation between the cerebellar hemispheres. He deteriorated clinically to a GCS of 8 and with intensive care support was intubated with rapid sequence induction.

There was no known travel history and polymerase chain reaction (PCR) testing for meningococcal and pneumococcal causes done on admission were negative. A lumbar puncture was done demonstrating glucose in the CSF of 4.9mmol/L (plasma - 8.6mmol/L), CSF protein - 0.79g/L. Microscopy of the CSF showed <1 mono, 6 poly and 1 red blood cells with no organisms and no growth after 48 hours. The opening pressure was 20 cm H20. Biofire screening was negative. The viral serology screen was negative. A vasculitic screen demonstrated both antinuclear antibody (ANA) and antineutrophil cytoplasmic antibody (ANCA) being negative, C3 1.22g/L, C4 0.20g/L, IgA 1.27g/L, IgG 7.70g/L, IgM 0.97g/L and electrophoresis with nil paraprotein band seen. Tuberculosis PCR was negative and acid-fast bacilli were not seen. The screening was negative for syphilis and serology was negative for leptospirosis and Lyme’s/tick-borne encephalitis.

**Figure 2:**
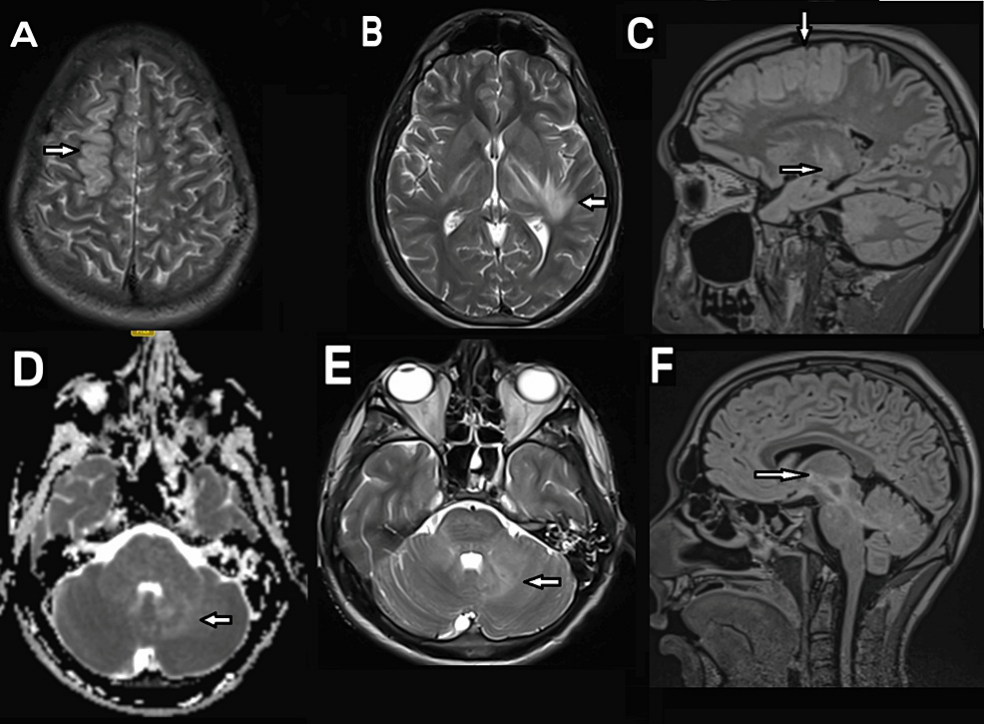
MRI with contrast with arrows demonstrating multifocal involvement of the right superior frontal gyrus (A and C), posterior limb of left internal capsule and subcortical U-fibres in the adjacent temporal lobe (B and C), cerebellum (D and E), and brainstem (F)

Due to the patient's impaired neurological status, we sought an MRI (Figure 2). This was markedly abnormal with cortical, cerebellar peduncular and deep tract brainstem involvement. 

**Figure 3:**
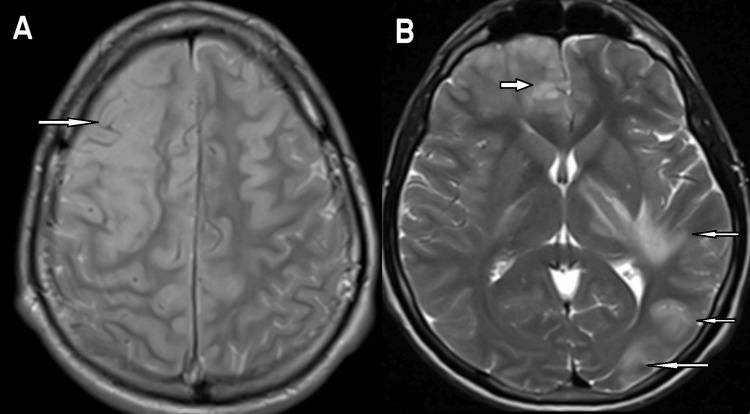
T2 weighted images of the frontal lobe demonstrate increased T2 signal (arrows) in both frontal lobes, the posterior limb of the left internal capsule and subcortical U-fibres in the adjacent temporal lobe

This was repeated a few days later with the MRI (Figure 3) demonstrating the evolution of the disease process which was not diagnostic but there was a vascular territory and cortical involvement which raised the possibility of vasculitis. Some foci of disease were new, (e.g., occipital), but a substantial extension of prior abnormalities was seen in the right frontal lobe.

Toxicology advice was sought, subsequently a urine sample was tested and demonstrated a THC metabolite. Synthetic cannabinoids (SCRAs) were then tested but the sample was negative for the six commonly seen SCRAs (5F-MDMB-PICA, 4F-MDMB-BINACA, MDMB-4en-PINACA, ADB-BUTINACA, 5F-ADB, AMB-FUBINACA).

After a multi-disciplinary meeting to discuss this case, the patient was commenced on IV methylprednisolone. The patient responded and was slowly weaned off sedation and was found to be less decerebrate, breathe spontaneously, and not require inotropic support after which he was weaned onto a tracheostomy. Once the course of methylprednisolone was completed, he transitioned onto a weaning dose of prednisolone.

Unfortunately, he became unwell again with agitation, an increasing oxygen requirement and a rise in liver transaminases which necessitated the recommencement of inotropic support and eventually, reintubation. A CT thorax delineated a tension pneumothorax as well as an empyema which was thought to be secondary to aspiration whilst this patient was intubated. Klebsiella was grown following a CT-guided biopsy and the empyema drained with a chest drain and then treated with a prolonged course of antibiotics, after which he was weaned off the ventilator once more. 

**Figure 4:**
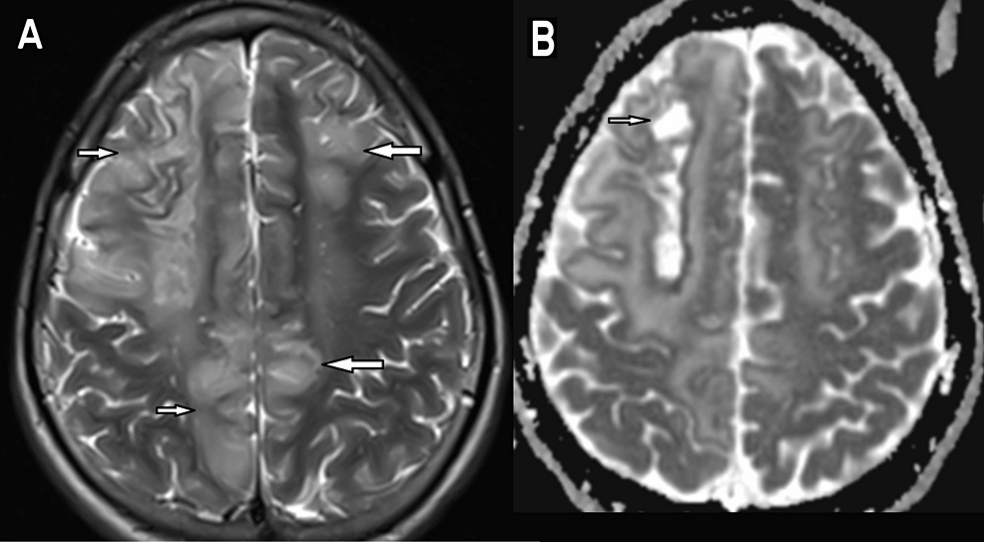
(A) T2-weighted imaging demonstrating post-inflammatory resolution with residual high signal; (B) Apparent Diffusion Coefficient (ADC) map normal diffusion facilitation

A third MRI (Figure 4) with contrast showed improved scan changes, significant gyriform contrast enhancement as well as bilateral basal ganglia and middle cerebellar peduncle T2 hyper intensity. Clinically, the patient was noted to have severe profuse sweating episodes, hyperthermia, hypertension and tachycardia. A query of seizures was raised, and a subsequent EEG carried out demonstrated diffuse background slowing in both hemispheres but no diagnostic focal or generalised epileptiform activities, PLEDS or triphasic activity. There was no evidence of ongoing seizures or non-convulsive status epilepticus episodes. As a result, he was diagnosed with PAID syndrome thought to be secondary to the severe brain injury sustained due to ANE.

On discharge, he had a GCS of 14/15 but was able to follow commands; for instance, squeezing and releasing with his right hand. There was receptive dysphasia in following 2-part commands and reduced cognition as he did not recall who he lived with at home previously. He complained of reduced vision for which glasses helped somewhat and he had poor truncal and neck control. He required the assistance of two people for all care and transfers as well as being doubly incontinent.

He was transferred to a rehabilitation hospital for further physiotherapy support where it was noted that he had weakness in his proximal muscles and his lower limbs. A nerve conduction study demonstrated evidence of myopathic changes in the upper limbs indicative of critical illness neuro-myopathy together with common peroneal nerve dysfunction. Both of these findings were likely secondary to his prolonged stay in intensive care. A further contributing factor to his myopathy could be steroid-induced myopathy secondary to high-dose treatment used in the acute phase of the illness. The culmination of these factors has left him significantly neurologically impaired [5]. 

## Discussion

ANE is a challenging diagnosis to make partly due to a combination of the rarity of the condition as well as its non-specific clinicoradiological presentation. Two-fifths of patients have convulsions, 28% exhibit impaired consciousness and 20% demonstrate emesis. Motor deficits such as spasticity, intention tremor, choreoathetosis, speech impairment, ataxia, visual disturbances, and other focal neurologic signs develop in the later chronic stages (as in this case) [6,7]. Also noteworthy is the presence of elevated transaminases in the acute phase present in approximately 62% of patients (also illustrated in this case) [8].

The pathogenesis of the disease is due to the presence of high cytokine levels [2]. However, scientists have previously identified a genetic abnormality in the variance of the RANBP2 gene which is associated with familial ANE. This indicates that in a proportion of patients, a genetic factor predisposes to encephalopathy in the setting of a prodromal event. However, the precise mechanism remains unknown with Levine et al. proposing that it may be due to dysfunctions in nucleocytoplasmic trafficking and intracellular metabolic regulation, as well as cytokine storm, and abnormal distribution of mitochondria [8].

Due to the rarity of ANE, studies which investigate potential therapeutics are few. However early introduction of steroids (within 24 hours after onset) has been associated with improved outcomes, with the proposed mechanism believed to be in treating the cytokine storm and metabolic dysfunction by decreasing inflammation. Additionally, the use of high-dose steroids may also limit the development of cytotoxic oedema associated with neuroinflammation [9]. Positive effects have not been obtained with intravenous immunoglobulin (IVIG), antivirals, or plasmapheresis as they do not mitigate the underlying pathology of the condition [9].

Although studies are limited, one potential avenue of further research may be the use of monoclonal antibodies directly targeting the proliferation of interleukin-1, interleukin-6 and tumour necrosis factor receptor-1. As they are the main proinflammatory cytokines of the cytokine storm that underlies ANE, inhibiting these may limit the downstream cytokine activation and prevent secondary brain injury with Koh et al., 2019 demonstrating a benefit in the use of tocilizumab when used in conjunction with corticosteroids in a paediatric case [10].

PAID syndrome itself is a rare complication of a brain injury with symptoms including diaphoresis, hyperthermia, hypertension, tachycardia, and tachypnoea accompanied by hypertonic movement. The cyclic episodes can last minutes to hours and can persist for weeks or months after the initial insult [11]. Due to the nature of the symptoms, important differentials should be excluded urgently as they include sepsis, thyroid storm, neuroleptic malignant syndrome, malignant hyperthermia and cerebral herniation.

Whilst the exact physiology is uncertain, some evidence suggests that PAID syndrome develops due to the loss of GABAergic inhibition of cortical projections resulting in dystonic posturing [11]. Dysfunction of the diencephalic autonomic centres or their connections to other brain regions (subcortical, cortical and brainstem) leading to loss of inhibitory to sympathetic feedback loops can result in diaphoresis, hyperthermia, hypertension, tachycardia, and tachypnoea [12].

The basis of PAID treatment is managing dysautonomia and hypertonia. Medications such as morphine, beta-blockers, baclofen, bromocriptine, dantrolene, clonidine and gabapentin all have anecdotal evidence supporting their use [13]. In our case, the use of gabapentin and baclofen were used to good effect in controlling the adverse effects.

## Conclusions

Here we present the case of a 23-year-old male who developed ANE with the only possible precipitant found being THC rather than the typical cases of ANE being viral induced. Whilst he was treated with IV methylprednisolone and corticosteroids as in other reported cases, this gentlemen’s patient journey was complicated by multiple subsequent complications arising because of a prolonged hospital stay and the after-effects of ANE including PAID syndrome and an empyema which ultimately resulted in significant neurological morbidity.
